# Type of Inflammation Differentially Affects Expression of Interleukin 1β and 6, Tumor Necrosis Factor-α and Toll-Like Receptors in Subclinical Endometritis in Mares

**DOI:** 10.1371/journal.pone.0154934

**Published:** 2016-05-06

**Authors:** Marta J. Siemieniuch, Anna Z. Szóstek, Katarzyna Gajos, Roland Kozdrowski, Marcin Nowak, Kiyoshi Okuda

**Affiliations:** 1 Dep. of Reproductive Immunology and Pathology, Polish Academy of Sciences, Olsztyn, Poland; 2 Graduate School of Environment and Life Science Okayama University, Okayama, Japan; 3 EQUI-MEDICA Equine Practice, Chojnice, Poland; 4 Faculty of Veterinary Medicine, Wrocław University of Environmental and Life Science, Wrocław, Poland; 5 Faculty of Veterinary Medicine, Wrocław University of Environmental and Life Science, Wrocław, Poland; INSERM, FRANCE

## Abstract

Mares that fail to conceive or lose their embryos, without showing typical signs of clinical endometritis, should be suspected of subclinical endometritis (SE). In this study, the question was addressed: does SE fully activate selected mechanisms of innate immunity in mares? For this aim, expression of mRNAs for *Toll-like Receptor 2* and *4* (*TLR 2/4*), *interleukin 1β* (*IL-1β)*, *interleukin 6* (*IL-6)* and *tumor necrosis factor α (TNF)* was examined in control mares *versus* either mares suffering from chronic endometritis (ChE) or subacute suppurative endometritis (SSE). The concentrations of IL-1β, IL-6 and TNF-α in supernatants from endometrial tissue cultures after 4 h incubation were measured using the enzyme immunoassay (EIA) method. Eighty-two warmblood mares, of known breeding history, were enrolled in this study. Based on histopathological assessment, mares were classified as suffering from ChE, SSE or as being healthy. In addition, immuno-localization of both TLR2 and TLR4 as well as TNF-α was investigated in the equine endometria. The mRNA expression of *TLR2* (P < 0.01), *IL-1β* (P < 0.0001), *IL-6* (P < 0.0001) and *TLR4* and *TNF* (P < 0.05) was up-regulated in endometria of mares suffering from SSE compared with unaffected mares. Concentrations of IL-6 and TNF-α were increased only in mares exhibiting SSE, compared with unaffected (P < 0.01 for both) and ChE mares (P < 0.05 for both). Immuno-localization of TNF-α and TLRs was confirmed, both in unaffected and SE-affected endometria, and was present in the luminal and glandular epithelia and stromal cells. The severity of inflammation impacts the immune response and fosters activation of innate immunity mechanisms, as observed in the endometria of mares. The intracellular localization of TLRs and TNF-α in the endometria indicates a key role of endometrial epithelial and stromal cells in the immune response and inflammation.

## Introduction

Endometritis is one of the most important economic problems in both animal production and breeding horses for sport, because of seriously reduced reproductive efficiency. Endometrial infections are directly responsible for lowering conception rates, but also indirectly impair reproductive outcomes leading to early embryo losses, abortion, *placentitis* and delivery of intrauterine-infected foals [1]. A clinical form of endometritis can be easily diagnosed; however, a subclinical endometritis (SE) in mares is accompanied neither by fluid accumulation in the uterine lumen nor the presence of a vulvar discharge, and only occasionally very subtle irregularities can be observed during ultrasonography (USG) examination. Microorganisms, including pathogenic or opportunistic bacteria and fungi, and an inadequate immune response in mares, contribute equally to SE [1, 2]. Endometritis is most commonly associated with aerobic bacteria [3]. However, isolation of bacteria does not necessarily prove the presence of endometritis nor does failure to isolate bacteria exclude it [3–5]. In clinical cases, the most common strain isolated from the equine endometrium is β-hemolytic *Streptococcus spp*., followed by *Escherichia coli (E*. *coli)*, *Pseudomonas aeruginosa*, *Klebsiella pneumoniae*, *Staphylococcus spp*. and various fungi [1, 6, 7]. Fifty-five percent of mares that were accumulating fluid in the uterus were bacteriologically positive, and in 81% of those mares *Streptococcus zooepidermicus* (*S*. *zooepidermicus*) was isolated [8]. Besides being one of the most important agents causing clinical endometritis, *S*. *zooepidermicus*, which resides in deep endometrial glands in equine uteri, may lead to the development of subclinical, latent infections [9].

The contact of immune cells, including macrophages present locally in tissues, with invading microbes activates a cascade of immunological events, which are non-specific but strongly evolutionarily conserved reactions. Binding of bacterial wall components by Toll-like receptors (TRLs) leads to activation of a down-stream cascade, through the up-regulation of transcriptional factors, which in turn up-regulates transcripts for genes involved in the immune response, finally increasing synthesis and secretion of cytokines, chemokines, eicosanoids and antimicrobial peptide [10–12]. Cytokines, including interleukin (IL) 1β (IL-1β), IL-6, IL-10, tumor necrosis factor-α (TNF-α), chemokines, prostaglandins (PGs) and antimicrobial peptides, which act in an autocrine/paracrine manner, are secreted by cells expressing TLRs, such as activated immune cells. However, mucous membranes, including those within the endometrium, are endowed with several innate immune mechanisms to protect the female reproductive tract against infection [13–15]. In fact, TLR4 was shown to mediate a lipopolysaccharide (LPS)-challenge response in murine [14] as well as in bovine [16] epithelial and stromal endometrial cells. Moreover, the production and secretion of a pleiotrophic cytokine, TNF-α, was confirmed in cultured feline [17] and equine [18] endometrial cells. The severity of inflammation seems to be dependent on several factors, including bacterial strain, innate immunity and general condition of the female. In this report, we posed the question: does chronic endometritis (ChE) or subacute suppurative endometritis (SSE), which are different types of SE, activate selected mechanisms of innate immunity in mares with the same intensity? To address this hypothesis, we investigated (i) mRNA expressions of *TLR2* and *4*, *IL-1β*, *IL-6* and *TNF-α* in endometrial biopsies derived from control *versus* either ChE or SSE mares; (ii) concentrations of IL-1β, IL-6 and TNF-α in supernatants from endometrial tissue cultures; and (iii) immuno-localization of TLR2 and 4, and TNF-α in equine endometria.

## Material and Methods

### 2.1. Ethical approval for the use of animals

This study was approved by the II Local Ethics Committee in Wrocław (Wrocław University of Environmental and Life Sciences, Poland). Reference number of approval: 43/2011, date: 18 April 2011.

### 2.2. Animals and endometrial biopsy sampling

The material was collected from 67 warmblood mares suspected of SE (aged 6–23 years) and from 15 maiden mares not suspected of endometritis that served as a control group (young, aged 3–4 years, with no history of breeding), between February and September 2012 at a number of stud farms in the lower Silesia region of Poland (south-west Poland). Stud farms were located in the range of about 80 km from Wrocław (57’07N, 17’02 E) in Dziuplina, Książ, Oława, Strzegom and Wrocław. Uterine biopsies and blood samples were collected with animals' owners informed consent.

Criteria for mares to be enrolled in the SE study were that they had been bred three or more times unsuccessfully in the breeding season, or had a history of ≥ one year of reproductive failure. None of the mares was in foaling heat, additionally none of the mares included in the study showed fluid in the uterus and involution of the uterus was completed. None of the mares had dystocia, retained fetal membranes or problems during puerperium. A blood sample was collected from the jugular vein of each mare. All mares were examined by transrectal palpation and USG (Honda HS-1500V) for genital tract evaluation and determination of estrous cycle stage and by measurement of serum progesterone (P_4_) level [19–21], as described in previous studies [22, 23]. None of the mares included in the study showed fluid in the uterus, so that any mares suffering from clinical endometritis were not enrolled in this study. Thirty-six mares were in estrus and had a dominant follicle, and 46 mares were in diestrus and had a corpus luteum (CL). Blood samples were kept refrigerated until centrifuged (1500 × *g* for 20 min) and pipetted to collect serum. Serum was stored at −20°C until assayed. Progesterone concentrations were determined using a commercial Progesterone ELISA kit (ENZO Life Sciences Inc., Farmingdale, NY, USA; ADI-901-011).

Endometrial biopsies (EB) were collected as already described [22]. Briefly, a sterilized biopsy punch was used (Equi-Vet, Kruuse; Denmark). After USG examination, the tail was bandaged, and then the vulva and perineum were cleaned with iodopovidone (Betadine, EGIS, Warsaw, Poland), rinsed three times with water, and dried with a paper towel. The instrument was passed through the vagina and cervix into the uterus with a sleeved and lubricated arm. After the forceps were placed in the uterine lumen, the arm was withdrawn from the vagina and inserted into the rectum to guide the forceps to the desired location. The uterine biopsy was obtained from the base of the uterine horn. Samples of EB were immediately smeared onto culture media for microbiological examination as described in a previous report [22]. Afterwards, each EB was divided with sterile scissors and forceps into three pieces. One piece was fixed in 4% formaldehyde for histopathological examination, while the second piece was plunged into RNAlater (Sigma Aldrich, R0901) for further analysis of gene expression, and the third piece was plunged into Dulbecco's Modified Eagle's Medium (DMEM) without phenol red (Sigma Aldrich, D6429) and transported to the laboratory at 4°C, all within 4 h after sampling.

### 2.3. Histology

Endometrial biopsies were fixed in 4% formaldehyde and stained routinely with hematoxylin and eosin or used for immunohistochemical staining. Biopsy specimens were evaluated by light microscopy according to Kenney and Doig [24] for classification of fibrosis grade *(endometrosis*, or chronic degenerative endometritis). Because some of the mares included in the study were barren for one year or more, and may have had chronic inflammatory changes with lymphocyte infiltration, evaluation of inflammation was based on counting both mononuclear and polymorphonuclear neutrophil (PMN) cell infiltration of the endometrial luminal epithelium and *stratum compactum*. This examination was based on the criteria described by Ricketts [25], Ricketts and Alonso [26] and Nielsen [27]. Briefly, if the *stratum compactum* was densely infiltrated by mononuclear cells, it was considered as a chronic endometritis (ChE), and if two or more PMNs per five fields (400 x magnification) were observed together with mononuclear cell infiltration the sample was considered as a subacute suppurative endometritis (SSE) [28, 29].

A total of 45 (54.8%) of the mares were positive for endometritis and 67 (81.7%) for periglandular fibrosis. Twenty-two mares with no inflammatory cells indicating endometritis on histological examination, but suffering from fibrosis (2A or 2B), were excluded from this study. Judging by the type of inflammation present, a total of 60 mares was assigned into 3 groups. Fifteen mares aged 3–4 years were in the control group and exhibited no history of breeding, no endometritis detected in histological examination and no fibrosis. Another 32 mares exhibited ChE and 13 more showed SSE.

### 2.4. Experiments

For Experiment 1, EBs were obtained from maiden, non-affected mares serving as a control (n = 15), mares suffering from ChE (n = 32) and those with SSE (n = 13). The control group consisted of mares exhibiting neither endometritis nor fibrosis in histological examination. The determination of *IL-1β*, *IL-6*, *TNF-α*, *TLR2* and *TLR4* mRNA transcription in the endometrial samples was performed using real-time PCR. For the second part of Experiment 1, *ex vivo* endometrial tissue cultures were prepared, with subsequent measurement of IL-1β, IL-6 and TNF-α concentrations in the culture supernatants were performed. The endometrial explants, directly after collection, were plunged into 1 mL DMEM and held at 4°C, then transported to the laboratory. The time between biopsy collection and the start of incubation (3–4 h) served as an acclimation period. Then, endometrial pieces were slightly desiccated, weighed and placed into fresh sterile glass tubes followed by addition of 1 mL fresh DMEM medium at 37°C. Endometrial tissue cultures were placed in a shaker water bath for 4 h at 37°C in a gas mixture (5% CO_2_ in air). After 4 h of incubation, the culture media, in amounts of 1 mL from each glass tube, were collected and frozen at -20°C until IL-1β, IL-6 and TNF-α measurements. The incubation time used in this study was based on our earlier experiments on spontaneous PGs and leukotrienes secretion in *ex vivo* endometrial tissue cultures from mares suffering from SE [23]. Cytokine concentrations, obtained from all those previous measurements, were calculated per one gram of tissue.

For Experiment 2, endometrial biopsies were obtained from maiden, unaffected mares serving as a control (n = 15), mares suffering from ChE (n = 32) and those with SSE (n = 13), similar to Experiment 1. The TNF-α, TLR2 and TLR4 protein immuno-localization in the endometrial samples was performed using immuno-histochemical (IHC) staining. Immunostaining against endometrial TLR2, TLR4 and TNF-α was done according to published protocols [15, 17]. Fragments of endometrial biopsies that had been fixed in 4% formaldehyde were embedded in paraffin, cut on a microtome at 3–4 μm slices and placed onto slides. For deparaffinization and rehydration, slides were placed into xylol followed by a graded series of alcohol: 100%, 96% and 70%. Antigen retrieval was performed by cooking (3 x 5 min) in citrate buffer (10 mM, pH = 6) in a microwave oven at 560 W and then cooling at 20°C for 20 min. For endogenous peroxidase activity blocking and background staining reduction, sections were placed for 30 min into a mixture of methanol and H_2_O_2_. Non-specific sites were blocked by adding 10% goat serum. Slides were incubated overnight at 4°C with the primary antibody for TLR2 (1:300, polyclonal rabbit anti-human CD282 (# AHP1424), AbD serotec, Toronto, Canada), TLR4 (1:600, rabbit polyclonal anti-TLR4 antibody (# ab13556), Abcam, Cambridge, MA, USA), and TNF-α (1:1500, rabbit polyclonal TNF-α antibody (# ab19139), Abcam). As a second antibody, biotinylated goat IgG against rabbit immunoglobulin (Vector Laboratories, Burlingame, CA, USA, # BA-1000, at a dilution of 1:100) was used for both TLRs and TNF-α. To visualize signals, slides were incubated with the substrate 3,3’ diaminobenzidine (DAB; DakoCytomation, Glostrup, Denmark) until a brown color appeared, and to obtain a contrast, sections were counterstained with hematoxylin. After washing under tap water, the slides were consolidated in Histokit (Assistent, Osterode, Germany).

### 2.5. Real-Time Polymerase Chain Reaction

TRI-reagent® (Sigma Aldrich, St. Louis, MO, USA, # T9424) was used to isolate total RNA according to the method of total RNA isolation by a single extraction with an acid guanidinium thiocyanate-phenol-chloroform mixture as described by Chomczynski and Sacchi [30]. The RNA was then quantified using the Nanodrop system (ND200C; Fisher Scientific, Hampton, PA, USA). The ratio of absorbance at 260 and 280 nm (A260/280) was approximately 2. The RNA (1 μg) was reverse transcribed into cDNA in a 20 μL reaction volume using oligo(dT) primer with a QuantiTect Reverse Transcription Kit (Qiagen, Hilden, Germany, # 205314), according to the manufacturer’s instructions, as already described [31]. The cDNA was stored at -20°C until real-time PCR was carried out. The primers used in the present study are listed in Table 1.

**Table 1:** List of factors analyzed, sequences of PCR primers used for expression studies, product sizes, accession numbers and references.

Gene name	Primer sequence (5’–3’), forward/reverse	Amplicon length (bp)	Accession number	Reference
*IL-1β*	GGCCCAAAACAGATGAAGGGCA	90	NM_001082526	Szóstek et al. 2013 [32]
	AAGTTGGTGGGAGAATTGAAGCTGG			
*IL-6*	GGATGCTTCCAATCTGGGTTC	221	NM_001082496.1	Szóstek et al. 2013 [32]
	CAGTTGGGTCAGGGGTGGTT			
*TNF*	ACCGAATGCCTTCCAGTCAA	143	AB035735	Galvao et al. 2013 [33]
	CATTTGCACGCCCACTCA			
*TRL-2*	GGCACTGGACCAGATCCTGAT	111	AY429602	Figueiredo et al. 2009 [34]
	TGGCATTCAGAGACCGAGAGA			
*TRL-4*	ATGCCCGTGCTGGGTTTTA	151	AY005808	Figueiredo et al. 2009 [34]
	ACTTTTTGCAGCCAGCAAGAA			
*SDHA*	GAGGAATGGTCTGGAATACTG	91	DQ402987	Cappelli et al.2008 [35]
	GCCTCTGCTCCATAAATCG			
*ACTB*	GGACCTGACGGACTACCTC	83	AF035774	Cappelli et al2008 [35]
	CACGCACGATTTCCCTCTC			
*GAPDH*	ATCTGACCTGCCGCCTGGAG	68	AF157126	Cappelli et al2008 [35]
	CGATGCCTGCTTCACCACCTTC			
*Β2M*	CCTGCTCGGGCTACTCTC	89	X69083	Capelli et al2008 [35]
	CATTCTCTGCTGGGTGACG			

Before running the assay, the reference gene was validated. To determine the most stable internal control gene, four potential reference genes were initially considered: *succinate dehydrogenase complex subunit A* (*SDHA*), *β-actin* (*ACTB*), *β-2-microglobulin (B2M)* and *glyceraldehyde-3-phosphate dehydrogenase (GAPDH)*. During the validation process, samples were run in parallel for the tested genes. The mRNA transcription of *SDHA* was the most stable and was unaffected by the experimental conditions (a less than twofold change between groups, as recommended by Dheda et al. [36]). Primer concentrations were optimized to the minimum concentration: lowest cycle threshold (Ct) ratio, and the final concentration was 500 nM per well. The assay was performed in a BioRad MyIQ (Bio-Rad Laboratories, Inc., Berkeley, CA, USA) using the default thermocycler program for all genes. Real-time PCR was carried out as follows: initial denaturation (10 min at 95°C), followed by 40 cycles of denaturation (15 sec at 95°C) and annealing (1 min at 60°C). After each PCR reaction, melting curves were obtained by stepwise increases in temperature from 60°C to 95°C to ensure single-product amplification. All reactions were performed in duplicate wells on a 96-well optical reaction plate (Multiplate PCR Plates, # MLP9601; Bio-Rad Laboratories) in a 10 μL reaction volume containing 2 μL water, 0.5 μL forward primer, 0.5 μL reverse primer, 5 μL SsoAdvanced SYBR Green Supermix (# 1725261; Bio-Rad Laboratories) and 2 μL cDNA (1 ng). Relative mRNA quantification data were then analyzed with the real-time PCR miner algorithm [37]. According to the instructions supplied for the miner algorithm (http://www.miner.ewindup.info/), after determination of average Ct and gene efficiency (E) for replicate samples, the Ct levels for each sample were related to the average E of a gene using the equation [1/(1+E)^^Ct^]. Thereafter, expression of the target genes was normalized against that of the *SDHA*. The presence of PCR product was also confirmed by electrophoresis on 2% agarose gel to check for size specificity of the amplicon (amplicons are listed in Table 1).

### 2.6. Cytokine measurement

For measuring IL-1β in supernatants from endometrial tissue cultures, the Equine IL-1β ELISA Reagent Set from GenWay Biotech, Inc. (# GWB-GIFBY0; San Diego, CA, USA) was used and run in accordance with the manufacturer's instructions. The standard curve for IL-1β covered the range 313 pg/mL to 20,000 pg/mL. The inter- and intra-assay coefficients of variation (CVs) were 11.9% and 8.9%, respectively.

For measuring IL-6 in supernatants from the endometrial tissue cultures, the GSI Equine IL-6 ELISA kit from Genorise Scientific, Inc. (# 106088; Glen Mills, PA, USA) was used and run in accordance with the manufacturer's instructions. The standard curve for IL-6 covered the range 81 pg/mL to 5200 pg/mL. Assay sensitivity was 16 pg/mL. The inter- and intra-assay CVs were 10.1% and 6.8%, respectively.

For measurement of TNF-α in supernatants from the endometrial tissue cultures, the Equine TNF-α DuoSet ELISA DEVELOPMENT SYSTEM (# DY1814; R&D Systems, Minneapolis, MN, USA) was used and run in accordance with the manufacturer's instructions. The standard curve for TNF-α covered the range 31.3 pg/mL to 2000 pg/mL. Assay sensitivity was 9.6 pg/mL. The inter- and intra-assay CVs were 12.1% and 7.8%, respectively.

### 2.7. Serum progesterone measurement

For serum P_4_ measurement, the Progesterone ELISA kit (ENZO Life Sciences Inc., Farmingdale, NY, USA; ADI-901-011) was used and run in accordance with the manufacturer’s instructions. The sensitivity of the P_4_ assay was 8.57 pg/mL. The cross-reactivity for a number of related steroids was as follows: P_4_ 100%, 5α-pregnane-3,20-dione 100%, 17-OH-progesterone 3.46%, corticosterone 0.77%, and deoxycorticosterone 0.28%. The inter- and intra-assay CVs were 8.1% and 9.5%, respectively.

### 2.8. Statistics

Data are shown as the mean ± SD of values obtained in separate experiments, each performed in duplicate. The values of endometrial *TLR2*, *TLR4*, *IL-1β*, *IL-6* and *TNF* mRNAs expression, and concentrations of IL-1β, IL-6 and TNF-α in supernatants from endometrial tissue cultures, were first tested to find if they fit a Gaussian distribution with the D'Agostino-Pearson omnibus normality test (GraphPad Software version 6, San Diego, CA, USA). The statistical analyses of endometrial *TLR2*, *TLR4*, *IL-1β*, *IL-6* and *TNF* mRNAs expression, and concentrations of IL-1β, IL-6 and TNF-α in the supernatants from endometrial tissue cultures after 4 h of incubation, were determined by nonparametric one-way ANOVA Kruskal–Wallis followed by Dunn’s test (GraphPad Software version 6, San Diego, CA, USA). Probability (P) values less than 0.05 were considered statistically significant.

## Results

### 3.1. Transcription levels of *IL-1β*, *IL-6*, *TNF* and *TLR2/4* genes in the equine endometrium and concentrations of IL-1β, IL-6 and TNF-α in the supernatant from endometrial tissue cultures

**Fig 1:**
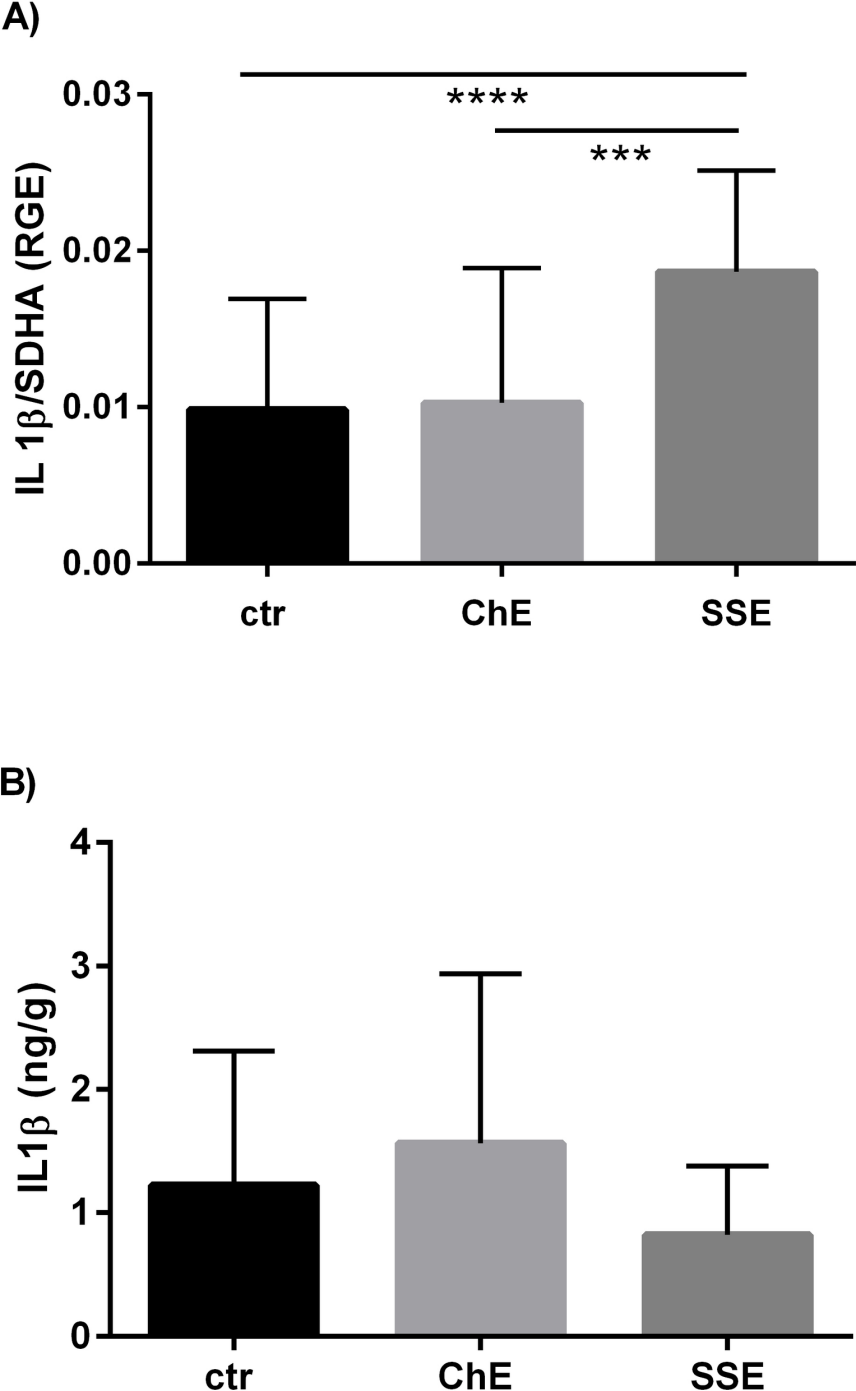
The mRNA level of *IL-1β* in the equine endometrium in relation to SE severity (A) and IL-1β concentration in the supernatant from endometrial tissue cultures in relation to SE severity (B). ctr—control mares, ChE—mares suffering from chronic endometritis, SSE—mares suffering from subacute suppurative endometritis. mRNA level of *IL-1β* and IL-1β concentrations in the supernatant are shown as means ± SD. Statistical significance was defined as values of P < 0.05. Statistical differences among groups are marked with asterisks (* P < 0.05, *** P < 0.001, **** P < 0.0001).

The mRNA level of *IL-1β* in the equine endometrium and its concentration in the supernatant from endometrial tissue cultures are shown in Fig 1. Based on inflammation type, *IL-1β* mRNA was only up-regulated in SSE, when compared to ChE (P < 0.001) or control mares (P < 0.0001) (Fig 1A). The concentration of IL-1β in the supernatant was not affected by the severity of inflammation (Fig 1B).

**Fig 2:**
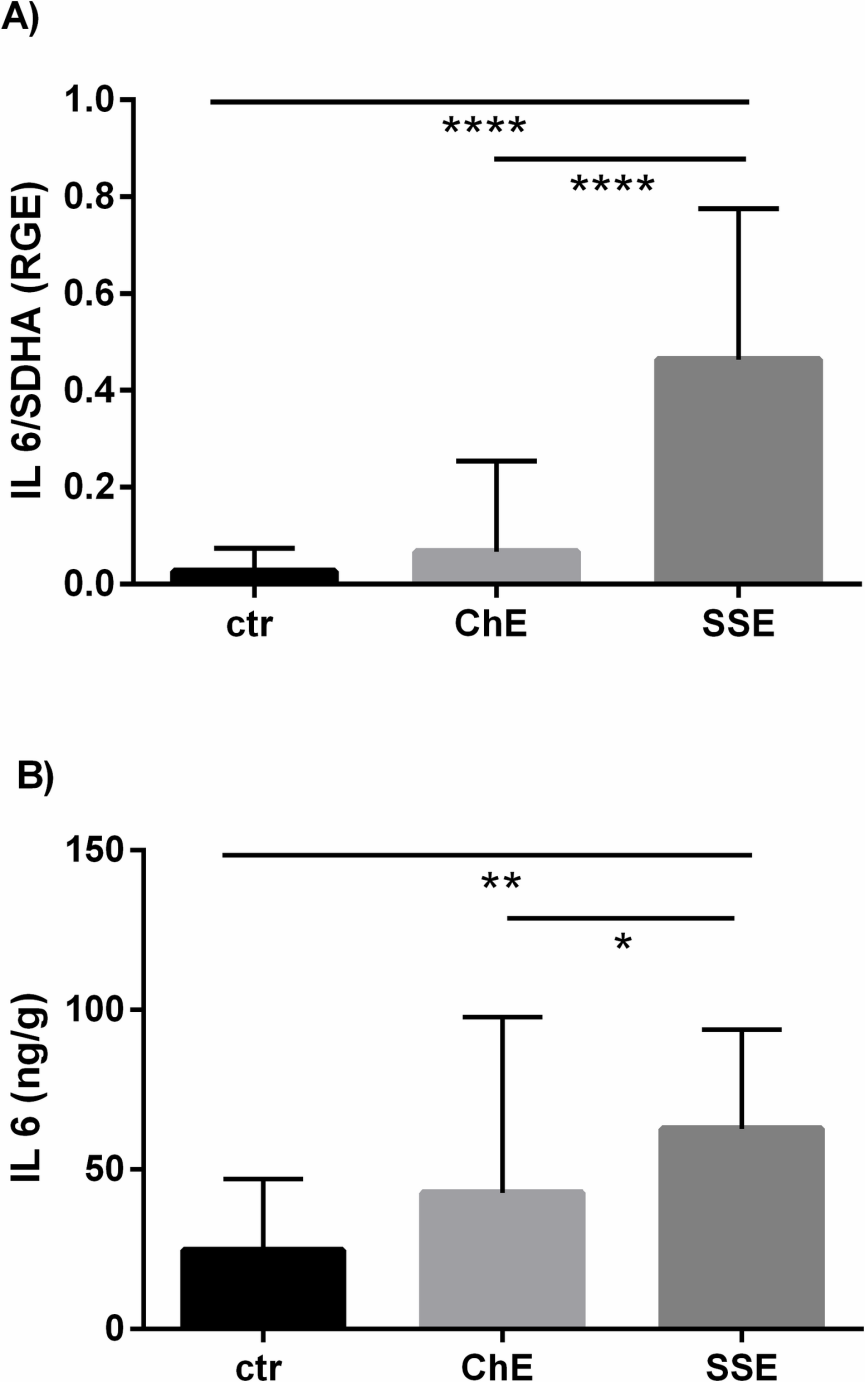
The mRNA level of *IL-6* in the equine endometrium in relation to SE severity (A) and its concentration in the supernatant from endometrial tissue cultures in relation to SE severity (B). ctr—control mares, ChE—mares suffering from chronic endometritis, SSE—mares suffering from subacute suppurative endometritis. mRNA level of *IL-6* and IL-6 concentrations in the supernatant are shown as means ± SD. Statistical significance was defined as values of P < 0.05. Statistical differences among groups are marked with asterisks (* P < 0.05, ** P < 0.01, **** P < 0.0001).

The mRNA level of *IL-6* in the equine endometrium and its concentration in the supernatant from endometrial tissue cultures are shown in Fig 2. Expression of *IL-6* mRNA was up-regulated only in SSE, compared to ChE as well as to control mares (P < 0.0001) (Fig 2A). The pattern of IL-6 concentrations in the medium was similar to *IL-6* mRNA expression. The concentration of IL-6 in the supernatant was the greatest in SSE, when compared to ChE (P < 0.05) or unaffected mares (P < 0.01) (Fig 2B).

**Fig 3:**
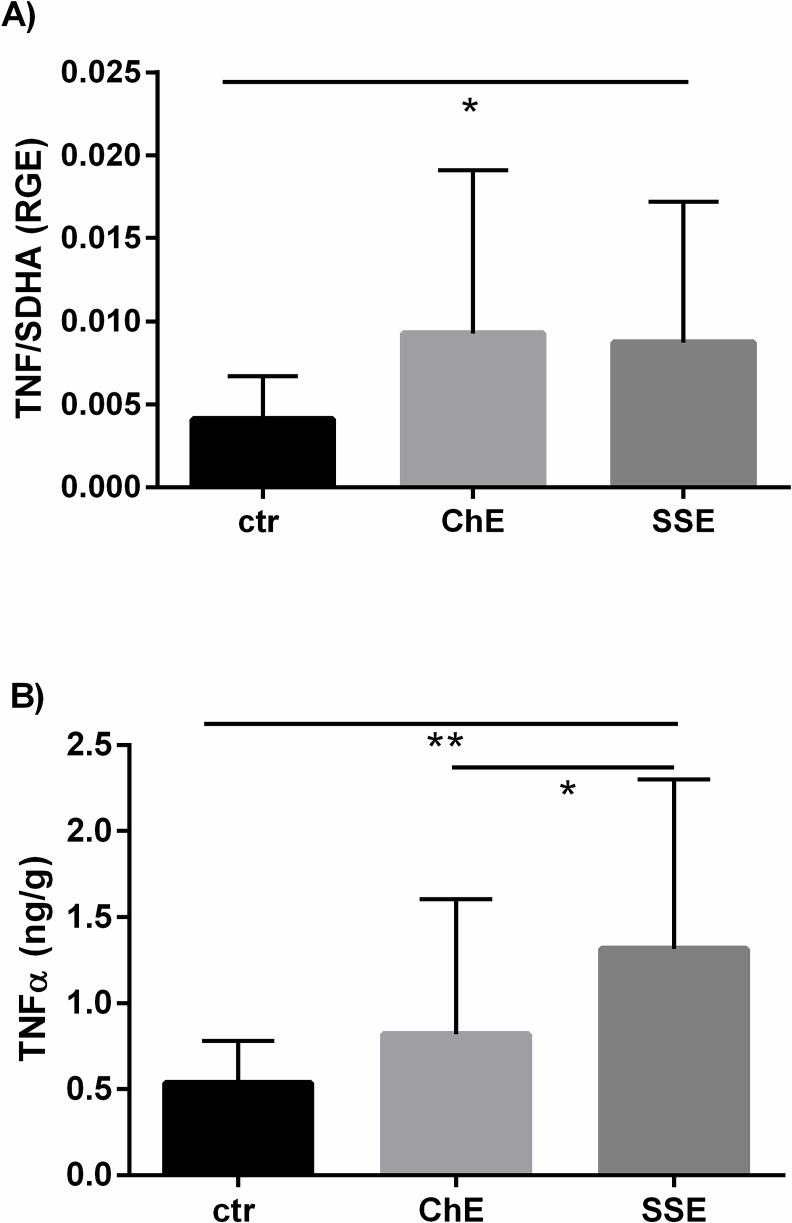
The mRNA level of *TNF* in the equine endometrium in relation to SE severity (A) and its concentration in the supernatant from endometrial tissue cultures in relation to SE severity (B). ctr—control mares, ChE—mares suffering from chronic endometritis, SSE—mares suffering from subacute suppurative endometritis. mRNA level of *TNF* and TNF-α concentrations in the supernatant are shown as means ± SD. Statistical significance was defined as values of P < 0.05. Statistical differences among groups are marked with asterisks (* P < 0.05, ** P < 0.01).

The mRNA level of *TNF* in the equine endometrium and its concentration in the supernatant from endometrial tissue cultures are shown in Fig 3. The mRNA level of *TNF* was up-regulated in both SE groups, when compared to control mares (P < 0.05) (Fig 3A). The concentration of TNF-α in the supernatant was the greatest in SSE compared to ChE (P < 0.05) or unaffected mares (P < 0.01) (Fig 3B).

**Fig 4:**
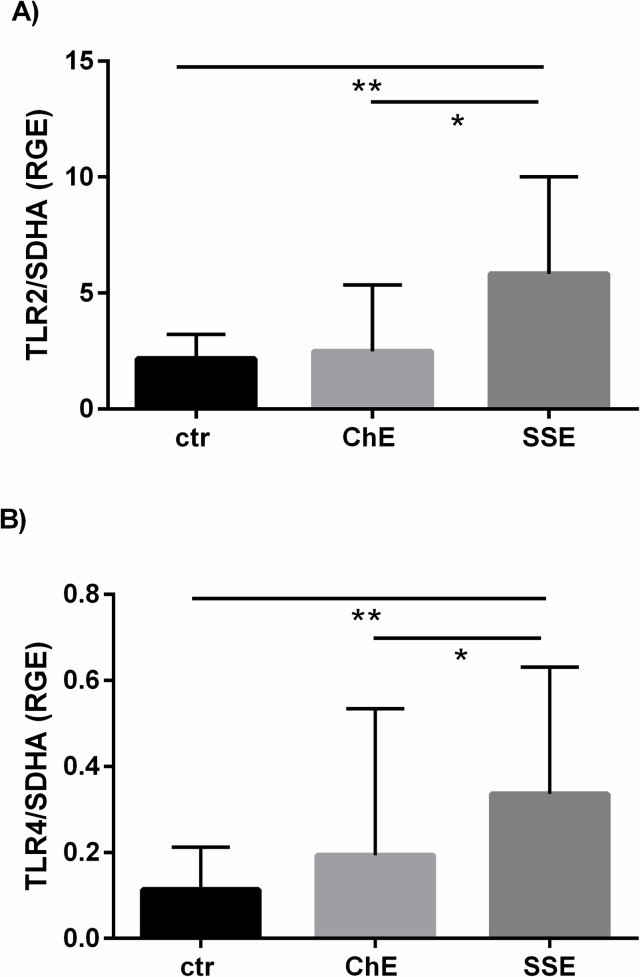
The mRNA level of *TLR2* (A) and *TLR4* (B) in equine endometrium in relation to SE severity. ctr—control mares, ChE—mares suffering from chronic endometritis, SSE—mares suffering from subacute suppurative endometritis. mRNA levels of *TLR2* and *TLR4* are shown as means ± SD. Statistical significance was defined as values of P < 0.05. Statistical differences among groups are marked with asterisks (* P < 0.05, ** P < 0.01).

The mRNA levels for both *TLR2* and *TLR4* in the equine endometrium are shown in Fig 4A and 4B, respectively). The mRNAs for both *TLR2* and *TLR4* were up-regulated in endometria of mares suffering from SSE, when compared to unaffected mares (P < 0.01 for *TLR2* and P < 0.05 for *TLR4*) or ChE (P < 0.05 for both).

### 3.2. Immuno-localization of TNF-α, TLR2 and TLR4

**Fig 5:**
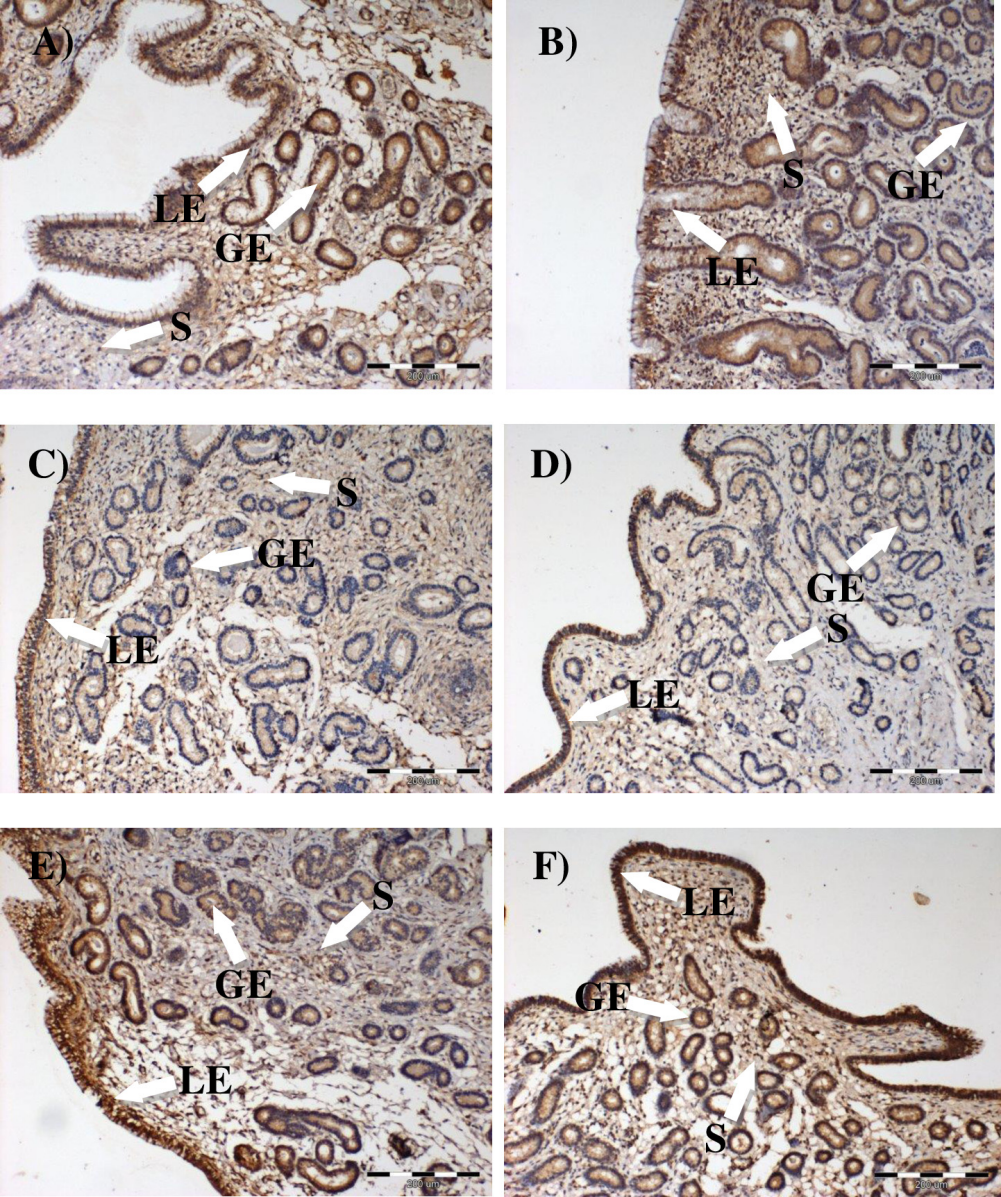
Immuno-localization of TNF-α (A, B), TLR2 (C, D) and TLR4 (E, F) in equine endometrial biopsies collected at estrus from control (A, C, E) or SE groups (B, D, F). LE-luminal epithelium; GE-glandular epithelium; S-endometrial stroma.

Immuno-localizations of both TLRs as well as TNF-α were confirmed in the luminal and glandular epithelia, and in endometrial fibroblasts, independently of the presence or absence of SE (Fig 5).

## Discussion

The pathophysiology of SE in mares remains poorly understood. Failure to conceive or early embryo loss, as well as altered length of the estrous cycle, are recurrent clinical signs of this condition. The immunological status of the mare and microbiological agents contribute to the pathogenesis of endometritis. There is increasing acceptance that during SE, innate immune mechanisms are not fully activated, which permits prolongation of inflammation and/or development of the latent form of inflammation [9]. It is also believed that chronic inflammation may inadequately activate several innate mechanisms; nevertheless, their prolonged activation, even if insufficient to eradicate infection, negatively affects the uterine microenvironment. This could further lead to perturbation in the expression of cytokines, metalloproteinases (MMPs), tissue inhibitors of metalloproteinases (TMPs), different types of collagen, fibronectin and hyaluronan [38].

This study describes alterations in the secretion of IL-1β, IL-6 and TNF-α, as well as changes in endometrial mRNA expression of *IL-1β*, *IL-6*, *TNF* and *TLRs*, during the course of SE, with respect to the severity of inflammation. In mares with endometritis, the concentrations of IL-6 and TNF-α in supernatants from endometrial tissue cultures were increased, compared with mares that did not suffer from endometritis, however, only in the cases of SSE and not ChE. In contrast, the concentration of IL-1β was unaffected either in ChE or in SSE. In cows, uterine inflammation was characterized by measurement of IL-6, IL-8, IL-1β, interferon (IFN) γ and TNF-α protein by ELISA in vaginal mucus derived from post-parturient cows presenting dystocia or eutocia [12]. Also, *ex vivo* endometrial tissue cultures, as well as *in vitro* endometrial epithelial and stromal cell cultures, were employed to show the complex contributions of inflammatory and tissue-damaging factors to endometritis [12]. The vaginal mucus results showed that the concentration of IL-6 was not increased, in contrast to IL-1β, the concentration of which increased in cows that had presented with dystocia three weeks earlier [12]. However, in another study describing the role of bovine endometrial epithelial and stromal cells in innate immunity *in vitro*, the concentrations of IL-1β were below the limits of detection of the assays for either LPS or lipopeptides [39]. The lack of IL-1β protein in the supernatant was explained by the complex process of IL-1β maturation, which is caspase-1 dependent, and pro-IL-1β cleavage to the active form is possible after formation of intracellular inflammasomes, which requires a second stimulus such as cell damage [39]. Therefore, it is assumed that some bacteria which affect endometrial cell survival may up-regulate caspase-1, and consequently lead to increasing concentrations of an active IL-1β. Indeed, lipopeptides of some lines of bacteria were shown to increase apoptosis [40]. It seems that tissue damage correlates with the severity of endometritis. Consequently, in acute clinical endometritis observed in cows suffering from dystocia that were clinically infected with Gram-positive *Trueperella pyogenes* (*T*. *pyogenes*) [12], the tissue damage was probably much more severe than in endometria of mares showing subclinical forms of endometritis. However, in another part of that study, using endometrial tissue cultures and *in vitro* endometrial cell cultures, LPS stimulated the secretion of IL-6 in all cultures [12]. This observation is in accordance with the present results, in which the concentration of IL-6 in endometrial tissue cultures was increased in SSE. Even if the secretory activity of bovine endometrial tissue cultures challenged with LPS is partially in accordance with our results, it is intriguing that Gram-negative bacteria were infrequently isolated from the cases of equine SE enrolled in this study. Indeed, of the 82 endometrial biopsies collected from mares, and also used in the present study, 44 had positive microbiology, however, *E*. *coli* was cultured from only 4 biopsies in our previous report [22]. These results concerning the low number of Gram-negative isolates is not surprising considering that most uterine infections in horses are caused by *Streptococcus spp*. In fact, *Streptococcus spp*. was cultured in 14 of 82 endometrial biopsies, while other Gram-positive bacteria, including *Bacillus spp*., *Corynebacterium spp*., *Staphylococcus spp*. and *Micrococcus spp*., were isolated from another 16 biopsies [22]. These discrepancies in secretory endometrial responses, observed in both cows and mares, may be due to different types of endometritis. Indeed, the clinical form was observed in cows while in the present study a subclinical type was found in mares. Moreover, species-specific differences in emergence of endometritis are also a possible explanation for this observation. In fact, post-parturient uterine inflammation is frequently observed in species in which tissue damage takes place during parturition and, in this case, bacteria localized in the lower genitourinary tract may ascend to the uterine lumen through the cervix [12, 40, 41]. In the present study, which was done in mares, the commencement of endometrial infection was definitely not a result of puerperial endometritis as reported in cattle. We assume that the unchanged IL-1β concentrations in supernatants from organ endometrial culture in mares may be due to the complex course of this disease, which often proceeds chronically and does not cause distinctive tissue damage, in contrast to clinical endometritis in which IL-1β secretion is up-regulated. Nonetheless, in another study [42], IL-6, IL-1β and TNF-α levels were investigated in menstrual effluents of women with chronic endometritis. Furthermore, a logistic regression analysis provided evidence that the IL-6/TNF-α-based model, excluding IL-1β, is a significant predictor of chronic endometritis [42], which is in accordance with the present results.

In the present study, an over-expression of mRNAs for *IL-1β*, *IL-6* and *TNF* in SSE mares was observed. This is in agreement with results of molecular studies performed on the endometria of cows, in which mRNAs for both *IL-1β* and *IL-6* were over-expressed during the course of endometritis [13, 43]. However, information concerning disturbances in the endometrial immune-endocrine balance during equine SE is still sparse and mostly dedicated to the clinical form of endometritis, which develops after breeding and presents an acute, short time-course [5, 44–47] in contrast to the subclinical and typically chronic time course of SE.

Cytokines or interleukins are secreted not only by activated immune cells, but also by endometrial cells that express TLRs. In this study, TLR2 and TLR4 protein expression was confirmed in equine endometrial epithelial and stromal cells. Furthermore, up-regulation of both *TLR2* and *TLR4* gene expression was found in the endometria of mares suffering from SSE but not in ChE. In bovine endometrial epithelial and stromal cells *in vitro*, increasing concentrations of IL-6 were found in response to lipopeptides released from Gram-positive bacteria, and this effect was abolished by providing small interfering RNA (siRNA) targeting *TLR2* and *TLR1* [39]. Intriguingly, that study showed that sex steroids indiscriminately affect the inflammatory response to lipoprotein [39]. Moreover, that study also showed that endometrial epithelial and stromal cells can respond to bacteria directly, not only through immune cells. This seems to be true also for equine endometrium, in which we confirmed the immuno-localization of TLR2 and 4 in different types of cells, including epithelial and stromal cells. Furthermore, Szóstek and coworkers [32] confirmed IL-1β and IL-6 protein expression in the endometrial epithelial and stromal cells of mares presenting 1, 2A/B and 3 categories of fibrosis. Since this immuno-histological study was performed by our group, we decided not to repeat IL-1β and IL-6 staining. Nevertheless, the present study confirmed expression of TNF-α protein in the endometrial superficial and glandular epithelium, as well as in stromal cells. This is in agreement with the study of Szóstek and coworkers [18], in which the direct production of TNF-α by *in vitro* cultured equine endometrial epithelial and stromal cells was shown by the ELISpot method or by IHC [33].

To conclude, the grade of inflammation is assumed to affect the severity of immune response during the course of chronic subclinical endometritis measured by the concentration of interleukins and cytokine in supernatants from *ex vivo* endometrial tissue cultures and by expression of genes for the respective factors as well as TLR2 and TLR4 proteins. In particular, during chronic and poorly expressed inflammation, immune non-specific mechanisms may not be activated properly, as needed to support a recovery from infection. The endometrial epithelial and stromal cells may contribute equally to an immune response against pathogens, due to intracellular localization of TLRs as studied here and to cytokine/interleukin proteins in the endometrium of mares.

## References

[1] LeBlanc MM, Causey RC. Clinical and subclinical endometritis in the mare: both threats to fertility. Reprod Dom Anim. 2009;44: 10–22. 10.1111/j.1439-0531.2009.01485.x 19660076

[2] Overbeck W, Witte TS, Heuwieser W. Comparison of three diagnostic methods to identify subclinical endometritis in mares. Theriogenology 2011;75: 1311–1318. 10.1016/j.theriogenology.2010.12.002 21251703

[3] Riddle WT, LeBlanc MM, Stromberg AJ. Relationships between uterine culture, cytology and pregnancy rates in a Thoroughbred practice. Theriogenology 2007;68: 395–402. 17583785 10.1016/j.theriogenology.2007.05.050

[4] Kenney RM. Cyclic and pathologic changes of the mare endometrium as detected by biopsy, with a note on early embryonic death. J Am Vet Med Assoc. 1978;172: 241–262. 621166

[5] LeBlanc MM. Advances in the diagnosis and treatment of chronic infectious and post-mating-induced endometritis in the mare. Reprod Domest Anim. 2010;45 Suppl. 2: 21–27. 10.1111/j.1439-0531.2010.01634.x 20591061

[6] LeBlanc MM, Magsig J, Stromberg AJ. Use of a low-volume uterine flush for diagnosing endometritis in chronically infertile mares. Theriogenology 2007;68: 403–412. 17543379 10.1016/j.theriogenology.2007.04.038

[7] Walter J, Neuberg KP, Failing K, Wehrend A. Cytological diagnosis of endometritis in the mare: Investigations of sampling techniques and relation to bacteriological results. Anim Reprod Sci. 2012;132: 178–186. 10.1016/j.anireprosci.2012.05.012 22727031

[8] Christoffersen M, Söderlind M, Rudefalk SR, Pedersen HG, Allen J, Krekeler N. Risk factors associated with uterine fluid after breeding caused by Streptococcus zooepidemicus. Theriogenology 2015;84: 1283–1290. 10.1016/j.theriogenology.2015.07.007 26300275

[9] Petersen MR, Skive B, Christoffersen M, Lu K, Nielsen JM, Troedsson MH, et al Activation of persistent Streptococcus equi subspecies zooepidemicus in mares with subclinical endometritis. Vet Microbiol. 2015;179: 119–125. 10.1016/j.vetmic.2015.06.006 26123371

[10] Takeuchi O. and Akira S. Pattern recognition receptors and inflammation. Cell 2010; 140: 805–820. 10.1016/j.cell.2010.01.022 20303872

[11] Cronin JG, Turner ML, Goetze L, Bryant CE, Sheldon IM. Toll-Like receptor 4 and MyD88-dependent signaling mechanisms of the innate immune system are essential for the response to lipopolysaccharide by epithelial and stromal cells of the bovine endometrium. Biol Reprod. 2012;86: 51–59. 10.1095/biolreprod.111.092718 22053092 PMC4396703

[12] Healy LL, Cronin JG, Sheldon IM. Endometrial cells sense and react to tissue damage during infection of the bovine endometrium via interleukin 1. Sci Rep. 2014;4: 7060 10.1038/srep07060 25395028 PMC4231323

[13] Herath S, Lilly ST, Santos NR, Gilbert RO, Goetze L, Bryant CE, et al Expression of genes associated with immunity in the endometrium of cattle with disparate postpartum uterine disease and fertility. Reprod Biol Endocrinol. 2009;7: 55 10.1186/1477-7827-7-55 19476661 PMC2702306

[14] Sheldon IM, Roberts MH. Toll-like receptor 4 mediates the response of epithelial and stromal cells to lipopolysaccharide in the endometrium. PLoS One 2010;5: e12906 10.1371/journal.pone.0012906 20877575 PMC2943929

[15] Jursza E, Kowalewski MP, Boos A, Skarzynski DJ, Socha P, Siemieniuch MJ. The role of toll-like receptors 2 and 4 in the pathogenesis of feline pyometra. Theriogenology 2015;83: 596–603. 10.1016/j.theriogenology.2014.10.023 25481489

[16] Herath S, Fischer DP, Werling D, Williams EJ, Lilly ST, Dobson H, et al Expression and function of Toll-like receptor 4 in the endometrial cells of the uterus. Endocrinology 2006;147: 562–570. 16223858 10.1210/en.2005-1113 PMC2738982

[17] Jursza E, Szóstek AZ, Kowalewski MP, Boos A, Okuda K, Siemieniuch MJ. LPS-challenged TNFα production, prostaglandin secretion and TNFα/TNFRs expression in the endometrium of domestic cats in estrus or diestrus, and in cats with pyometra or receiving medroxyprogesterone acetate. Mediators Inflamm. 2014; 689280. 10.1155/2014/689280 PMC4083830 25028529

[18] Szóstek AZ, Adamowski M, Galvão AM, Ferreira-Dias GM, Skarzynski DJ. Ovarian steroid-dependent tumor necrosis factor-α production and its action on the equine endometrium in vitro. Cytokine 2014;67: 85–91. 10.1016/j.cyto.2014.02.005 24642167

[19] Pierson RA, Ginther OJ. Ultrasonic evaluation of the corpus luteum of the mare. Theriogenology 1985;23:795–806. 16726050 10.1016/0093-691x(85)90155-4

[20] Ginther OJ, Gastal EL, Gastal MO, Utt MD, Beg MA. Luteal blood flow and progesterone production in mares. Anim Reprod Sci. 2007;99: 213–220. 16815650 10.1016/j.anireprosci.2006.05.018

[21] Bergfelt DR, Adams GP. Luteal development In: McKinnon AO, Squires EL, Vaala WE, Varner DD, editors. Equine Reproduction. Blacwell Publishing Ltd; 2011, pp. 2055–2064.

[22] Buczkowska J, Kozdrowski R, Nowak M, Raś A, Staroniewicz Z, Siemieniuch MJ. Comparison of the biopsy and cytobrush techniques for diagnosis of subclinical endometritis in mares. RBE 2014;12: 27. 10.1186/1477-7827-12-27 PMC3986439 24708825

[23] Gajos K, Kozdrowski R, Nowak M, Siemieniuch MJ. Altered secretion of selected arachidonic acid metabolites during subclinical endometritis relative to estrous cycle stage and grade of fibrosis in mares. Theriogenology 2015;84: 457–466. 10.1016/j.theriogenology.2015.03.038 25963128

[24] Kenney RM, Doig PA. Equine endometrial biopsy In: Morrow DA, editor. Current Therapy in Theriogenology 2, Philadelphia, Pa Saunders WB; 1986, pp. 726–729.

[25] Ricketts SW. The technique and clinical application of endometrial biopsy in the mare. Equine Vet J. 1975;7: 102–107. 1140188 10.1111/j.2042-3306.1975.tb03243.x

[26] Ricketts SW, Alonso S. Assessment of the breeding prognosis of mares using paired endometrial biopsy techniques. Equine Vet J. 1991;23: 185–188. 1884698 10.1111/j.2042-3306.1991.tb02751.x

[27] Nielsen JM. Endometritis in the mare: a diagnostic study comparing cultures from swab and biopsy. Theriogenology 2005;64: 510–518. 15978661 10.1016/j.theriogenology.2005.05.034

[28] Schoon H.-A., Schoon D., Klug E., 1997 Die Endometriumbiopsie bei der Stute im klinisch-gynäkologischen Kontext. Pferdeheilkunde 13, 453–564.

[29] Kilgenstein H.J., Schöniger S., Schoon D., Schoon H.A.: Microscopic examination of endometrial biopsies of retired sports mares: An explanation for the clinically observed subfertility? Res. Vet. Sci. 2015, 99, 171–179. 10.1016/j.rvsc.2015.01.005 25639692

[30] Chomczynski P, Sacchi N. Single-step method of RNA isolation by acid guanidinium thiocyanate-phenol-chloroform extraction. Anal Biochem. 1987;162: 156–159. 2440339 10.1006/abio.1987.9999

[31] Szóstek AZ, Siemieniuch MJ, Łukasik K, Galvao AM, Ferreira-Dias GM, Skarżyński DJ. mRNA trancription of prostaglandin synthases and their products in the equine endometrium in the course of fibrosis. Theriogenology 2012;78: 768–776. 10.1016/j.theriogenology.2012.03.024 22578628

[32] Szóstek AZ, Lukasik K, Galvao AM, Ferreira-Dias GM, Skarzynski DJ. Impairment of the interleukin system in equine endometrium during the course of endometrosis. Biol Reprod. 2013;89: 79 10.1095/biolreprod.113.109447 23946535

[33] Galvão A, Valente L, Skarzynski DJ, Szóstek A, Piotrowska-Tomala K, Rebordão MR, Mateus L, Ferreira-Dias G. Effect of cytokines and ovarian steroids on equine endometrial function: an in vitro study. Reprod Fertil Dev. 2013;25: 985–997. 10.1071/RD12153 23075812

[34] Figueiredo MD, Salter CE, Andrietti AL, Vandenplas ML, Hurley DJ, Moore JN. Validation of a reliable set of primer pairs for measuring gene expression by real-time quantitative RT-PCR in equine leukocytes. Vet Immunol Immunopathol. 2009;131: 65–72. 10.1016/j.vetimm.2009.03.013 19376596

[35] Cappelli K, Felicetti M, Capomaccio S, Spinsanti G, Silvestrelli M, Supplizi AV. Exercise induced stress in horses: selection of the most stable reference genes for quantitative RT-PCR normalization. BMC Mol Biol. 2008;9: 49 10.1186/1471-2199-9-49 18489742 PMC2412902

[36] Dheda K, Huggett JF, Bustin SA, Johnson MA, Rook G, Zumla A. Validation of housekeeping genes for normalizing RNA expression in real-time PCR. Biotechniques 2004;37: 112–119. 15283208 10.2144/04371RR03

[37] Zhao S, Fernald RD. Comprehensive algorithm for quantitative real-time polymerase chain reaction. J Comput Biol. 2005;12: 1047–1064. 16241897 10.1089/cmb.2005.12.1047 PMC2716216

[38] Aresu L, Benali S, Giannuzzi D, Mantovani R, Castagnaro M, Falomo ME. The role of inflammation and matrix metalloproteinases in equine endometriosis. J Vet Sci. 2012;13: 171–177. 22705739 10.4142/jvs.2012.13.2.171 PMC3386342

[39] Turner ML, Cronin JG, Healey GD, Sheldon IM. Epithelial and stromal cells of bovine endometrium have roles in innate immunity and initiate inflammatory responses to bacterial lipopeptides in vitro via Toll-like receptors TLR2, TLR1, and TLR6. Endocrinology 2014;155: 1453–1465. 10.1210/en.2013-1822 24437488 PMC3959608

[40] Aliprantis AO, Yang RB, Mark MR, Suggett S, Devaux B, Radolf JD, et al Cell activation and apoptosis by bacterial lipoproteins through toll-like receptor-2. Science 1999;285(5428): 736–739. 10426996 10.1126/science.285.5428.736

[41] Sheldon IM, Cronin J, Goetze L, Donofrio G, Schuberth HJ. Defining postpartum uterine disease and the mechanisms of infection and immunity in the female reproductive tract in cattle. Biol Reprod. 2009;81: 1025–1032. 10.1095/biolreprod.109.077370 19439727 PMC2784443

[42] Tortorella C, Piazzolla G, Matteo M, Pinto V, Tinelli R, Sabbà C, et al Interleukin-6, interleukin-1β, and tumor necrosis factor α in menstrual effluents as biomarkers of chronic endometritis. Fertil Steril. 2014;101: 242–247. 10.1016/j.fertnstert.2013.09.041 24314919

[43] Gabler C, Fischer C, Drillich M, Einspanier R, Heuwieser W. Time dependent mRNA expression of selected pro-inflammatory factors in the endometrium of primiparous cows postpartum. Reprod Biol Endocrinol. 2010;8: 152 10.1186/1477-7827-8-152 21176181 PMC3016299

[44] Fumuso E, Giguère S, Wade J, Rogan D, Videla-Dorna I, Bowden RA. Endometrial IL-1beta, IL-6 and TNF-alpha, mRNA expression in mares resistant or susceptible to post-breeding endometritis. Effects of estrous cycle, artificial insemination and immunomodulation. Vet Immunol Immunopathol. 2003;96: 31–41. 14522132 10.1016/s0165-2427(03)00137-5

[45] Christoffersen M, Woodward E, Bojesen AM, Jacobsen S, Petersen MR, Troedsson MH, et al Inflammatory responses to induced infectious endometritis in mares resistant or susceptible to persistent endometritis. Vet Res. 2012;8: 41. 10.1186/1746-6148-8-41 PMC3368729 22458733

[46] Portus BJ, Reilas T, Katila T. Effect of seminal plasma on uterine inflammation, contractility and pregnancy rates in mares. Equine Vet J. 2005;37: 515–519. 16295928 10.2746/042516405775314844

[47] Woodward EM, Christoffersen M, Campos J, Betancourt A, Horohov D, Scoggin KE, et al Endometrial inflammatory markers of the early immune response in mares susceptible or resistant to persistent breeding-induced endometritis. Reproduction 2013;145: 289–296. 23580950 10.1530/rep-12-0452

